# Condom use education, promotion and reasons for condom use: Perspectives of healthcare providers and young adults in Vhembe district, Limpopo province

**DOI:** 10.4102/safp.v63i1.5326

**Published:** 2021-11-30

**Authors:** Namadzavho J. Muswede, Livhuwani Tshivhase, Azwihangwisi H. Mavhandu-Mudzusi

**Affiliations:** 1 Professional Practice, South African Nursing Council, Pretoria, South Africa; 2 Department of Nursing Sciences, Faculty of Health Care, University of Sefako Makgatho Health Sciences University, Pretoria, South Africa; 3 Department of Health Studies, College of Human Sciences, University of South Africa, Pretoria, South Africa

**Keywords:** approaches, promote, condom use, descriptive phenomenology, young adults

## Abstract

**Background:**

Condom use is a critical component of a comprehensive and sustainable approach to the prevention of unintended pregnancies and sexually transmitted infections (STIs) (including HIV). Despite government making condoms freely available in the healthcare facilities in Vhembe district, there are reports of an increase in teenage pregnancies and STIs, including HIV, amongst young adults. The aim of the study was to gain an in-depth understanding of condom use promotion and reasons of condom use amongst young adults in Vhembe district, in Limpopo province.

**Methods:**

A descriptive phenomenological design was used to explore the reasons for promoting condom use amongst young adults themselves and from the perspectives of healthcare providers who are critical role players in condom education and provision. Purposive sampling was used to sample young adults and healthcare providers at three of Vhembe district’s primary healthcare facilities. Individual semi-structured interviews were conducted, after which thematic data analysis was used to develop themes and subthemes.

**Results:**

Two superordinate themes emerged from data analysis, namely approaches to promote condom use and reasons for condom use. Two themes emerged in respect of approaches for promotion of condom use: information sharing in the form of education, the distribution of informative material, and the adoption of a multi-sectoral approach. Self-protection emerged as a reason for condom use, to prevent disease, pregnancy and ‘u wela’, and was indicative of not trusting a sexual partner.

**Conclusion:**

To effectively promote condom use, a multidisciplinary team approach involving nurses, lay counsellors and peer educators need to be strengthened at local primary health facilities in order to facilitate the distribution of condoms and educate young adults on consistent condom use.

## Introduction

Like many other countries in sub-Saharan Africa, South Africa faces challenges in reducing the incidence of new HIV infections. In 2020, the number of people living with HIV was estimated at approximately 7.8 million. Amongst those aged 15–49, approximately 18.7 % of the population is HIV positive.^1^ To reduce HIV infection rates, government introduced the Abstain, Be faithful and Condomise (ABC) strategy. The ABC strategy consists of three components: practising abstinence, being faithful to one sexual partner, and using condoms with all partners. This strategy remains the standard global recommendation used in sexually transmitted infection (STI) and HIV prevention programmes.^2^

Despite the above strategy, consistent condom use is, however, notably low, especially amongst people living with HIV in the 18–24-year age group. The situation is not much different in other sub-Saharan countries. Emmanuel, Neyole, Poipoi, Ringera and Otomu,^3^ who conducted a study focusing on condom use determinants and practices amongst people aged 18–24 years in Kenya, found that most study participants had the correct knowledge, in that they knew condoms are effective at preventing the spread of HIV; however condom use was notably low.

In Botswana, a study by Mashanda-Tafaune and Monareng^4^ on the perceptions and attitudes of healthcare workers towards using female condoms, found that the training of healthcare workers is essential for enhancing positive perceptions and attitudinal change with a view to reducing sexual risks, related infections and combatting poor quality of life amongst women. Healthcare providers’ negative attitudes were cited as one of the barriers preventing sexually active individuals from accessing condoms at primary healthcare facilities.^5^ These findings concur with those of Lebese, Maputle, Ramathuba and Khoza,^6^ who reported limited use of contraception amongst the Vatsonga in the Vhembe district, because of negative attitudes and poor health education on the part of service providers.

Studies, including those by Shisana, Rehle, Simbayi, Jooste, Zungu, Labadarios^7,8^ also revealed low condom use amongst vulnerable groups such as lesbian, gay, bisexual and transgender, queer and intersex (LGBTQI) communities, and people with disabilities. This is aggravated by the emerging and consistent evidence about the high HIV burden amongst men who have sex with men (MSM) in Southern Africa revealed by Bekker, Rebe, Venter, Maartens, Moorhouse, Conradie, Wallis, Harley, Black and Eakles^9^ and the fact that most MSM also have female sexual partners and they also engage in heterosexuality.

The National Adolescent and Youth Indicators report revealed high teenage pregnancies and termination of pregnancies amongst girls aged 10–19 years in Limpopo province. This situation is a problem for the country, with more than a million people treated for STIs in 2015. The National Strategic Plan on HIV, tuberculosis (TB), and STIs includes female condoms as a key component of its combination prevention package, but anecdotal evidence shows that female condom was generally not well received and therefore not maximally used as intended. In Vhembe district specifically, anecdotal evidence suggests a gradual decline in condom use and a rising number of STIs, with a concomitant increased HIV risk. A report by Sentinel Surveillance revealed about 1046–1932 new STIs amongst clients aged 18–20, at sampled facilities in the Vhembe district.^10^ Such a high number is a cause of concern in a district with a population of 1 393 949.12.^11^ These prompted the researchers to ask the following question: How are condom use promoted amongst young adults? Thus, this study focused on exploring condom use promotion and reasons of condom use amongst young adults in Vhembe district.

## Research methods

The researchers chose a descriptive phenomenology, as it is suitable for investigating a phenomenon in an in-depth and holistic fashion, through the collection of rich narratives. Descriptive phenomenology was chosen because it focuses on the lived experience of humans and the meanings they attach to these experiences in their own setting.^12^ The researchers aimed to gain a comprehensive understanding of condom use promotion and reasons of condom use amongst young adults in Vhembe district.

In the context of this study, evidence points to limited condom use and a general increase in the number of patients with STIs in the region under study. For that reason, the researchers sought to explore condom use promotion and reasons of condom use amongst young adults from the viewpoint of young adults and healthcare providers.

### Research setting

The study was conducted at three primary healthcare clinics in the Vhembe district, Limpopo province. The facilities were purposefully selected based on the evidence of monthly reporting of STI syndromes which were being increasingly prevalent amongst clients aged 18–20 years. In addition, the selected facilities were quite busy, with a head count average of between 4200 and 4600 per month as reported in Vhembe District Health Information System (DHIS) report.^11^

### Population

The study population were healthcare providers working in the primary health care (PHC) facilities and all young adults who visited the three sampled PHC facilities for a variety of health services in Vhembe district.

### Sampling

Purposive criterion sampling technique was used to recruit the participants. For the healthcare providers, the inclusion criteria included the following: working at the facilities during the data-collection period, working experience of more than 3 years in condom delivery service related activities.

This was to ensure that the data collected and analysed reflect some broad, comprehensive viewpoints on the phenomenon under study. The sample size for healthcare provider were nine (see Table 2), composed of the following categories: (1) Three of the healthcare providers were professional nurses who offered a comprehensive primary healthcare package, including HIV and **AIDS** prevention and promotion information. (2) Two were peer educators, whose primary function was to strengthen HIV or **AIDS** prevention by educating the public (especially the youth) on the importance of using condoms. They also distributed condoms to nearby hotspots such as taverns. (3) Two participants were community healthcare workers who had been trained to provide basic healthcare to the community; they went door to door and provided basic health education, promoted adherence to chronic treatment regimens (including anti-retroviral therapies [ARTs]), and reported or referred back to the clinic any patients who were not doing well or not complying with their treatment plans. While going door to door, they also distributed condoms. (4) Two participants were lay counsellors, based at the clinic, who provided pre- and post-HIV counselling and testing on all voluntary and provider-initiated testing, and referred clients appropriately, depending on the test results. Embedded in their primary function was health education or information sharing on the importance of condom use. All the healthcare providers who participated in the study had varying levels of experience in their field, ranging from 3 years to 10 years.

### Data collection

The primary researcher personally conducted all the interviews. A semi-structured interview schedule was used to guide the individual interviews that were held with recruited participants. The interview data were recorded using an audio recorder, which captured the participants’ verbatim responses, while affording the researcher an opportunity to listen carefully and direct the flow of the interview, in addition to taking field notes. Communication skills such as listening, probing, reflecting, clarifying and paraphrasing were used to gain a better understanding of the phenomenon under study.^12^ Sixteen interviews were conducted in Tshivenda and two were conducted in English as preferred by the participants. Data were collected from both groups of participants until saturation was achieved, meaning until there was no further or new information forthcoming from the participants.^12^ Saturation was reached at the 14th participant but two more participants from each group were interviewed to confirm the findings of the study. Sample size of 14 is justifiable by Polit and Beck^12^ who posit that in phenomenological studies, saturation can be achieved with six to twenty-five participants. In order to get more informative data, the researcher conducted the research with different categories of healthcare provider with three year’s experiences in condom delivery services. Because of staff shortages, in some instances the researcher had to wait for the participants (especially healthcare providers) to prioritise the rendering of services to clients, before participating in the interviews. The sampled facilities provided suitable spaces – a room and/or an office for conducting the interview sessions, depending on what was available.

### Data analysis

In qualitative research, data collection and analysis occur simultaneously when the researcher explores avenues mentioned by participants who may need clarity.^13^ However, comprehensive analysis was conducted immediately after data collection. The data were analysed manually, guided by the interpretative phenomenological analysis (IPA) framework described by Creswell.^13^ The researcher transcribed the audio recordings in detail. This included verbatim transcriptions of the participants’ comments, summary notes and self-reflecting notes captured in the course of the interviews. This ensured that the data being analysed would be correctly and sufficiently interpreted and contextualised. The data collected in Tshivenda was transcribed verbatim and then transcribed into English by a language specialist who was fluent in both Tshivenda and English. The researcher repeatedly read the texts, replayed the audio recordings, and re-examined non-textual data so as to order them, while familiarising and immersing herself in the data. The researcher took note of the words and phrases in the participants’ observations that captured the meaning they attached to their views^14^ as is briefly explained below:

- The researcher developed a list of significant statements by eliciting comments about how each participant experienced the topic. Significant statements were grouped into larger units of information to create meaning units or themes, which were further coded.^15^
- Next, the researcher put together themes after checking for connections or underlying similarities between them, and similar themes were clustered under one superordinate theme.^13^

Thereafter, the researcher developed a comprehensive table of superordinate themes, with themes and subthemes from the transcripts. Samples of interview records and all the tools used were subjected to transcription and further analysis by an independent researcher and coder (an expert in qualitative research), to ensure conformability. Sourcing the services of an independent coder on the same data helped to ensure reliability.^15^

## Trustworthiness

To ensure the trustworthiness of the study, as required in qualitative research approaches, the researcher employed four criteria as outlined by Lincoln and Guba (1985, cited in Polit and Beck).^12^ The researcher established a prolonged engagement of six months with the participants, dedicating time to them to ensure credibility, establish good relations and foster a sense of trust. An independent coder was used during data analysis to ensure the credibility of the study results and avoid bias. To ensure dependability, the researcher used semi-structured interviews with open-ended questions. Confirmability was ensured through quality audio recordings of the interviews, which were later transcribed and accompanied by field notes taken during the interviews. The independent coder analysed the data independently, before giving feedback to the researcher. The authenticity of the results was ensured through the inclusion of the participants’ verbatim quotes.

### Ethical considerations

Ethical approval for the study was granted by the Ethics Committee of the University of South Africa, prior to commencing data collection (HSHDC/583/2017). Permission was also sought from the Limpopo Provincial Department of Health (REC-012714-038-NHERC), the Vhembe district primary healthcare manager, and the operational managers of the primary healthcare facilities in question. For each study participant, the researcher ensured the following principles: informed consent, autonomy, justice, beneficence, non-maleficence, privacy and confidentiality when conducting the study. Informed consent was ensured through informing participants about their rights, purpose of the study, procedures, duration of the study, confidentiality of personal data and the voluntary participation to the study. Prior to the interviews, the participants were informed verbally and consent form were issued to be signed. In the reported findings, anonymity is ensured by using codes such as HCP for a participating healthcare provider, and P for a young participant, followed by a number.

## Results

### Biographical data

The young adult participants sampled were between the ages of 18 and 24 years (Table 1). The majority (7) were single, and two were married. Five participants had a secondary education, two had completed or were completing their tertiary education and two were in Grade 10. Six participants were female, and three were male. Except for one male, they were all unemployed. The second group of participants were healthcare workers (HCP), as displayed in Table 2. All health care workers interviewed were female and their descriptions are explained in the sampling section.

**TABLE 1:** Participants: Young adults.

Code	Age	Facility	Sex	Highest level of education	Marital status
P1	18	Facility C	Female	Grade 10	Single
P2	23	Facility A	Male	Tertiary	Single
P3	24	Facility A	Male	Grade 12	Married
P4	18	Facility B	Female	Grade 12	Single
P5	19	Facility B	Female	Grade 12	Single
P6	23	Facility B	Female	Grade 12	Married
P7	24	Facility A	Female	Grade 12	Single
P8	20	Facility B	Male	Tertiary	Single
P9	19	Facility A	Female	Grade 10	Single

Note: The second category of participants consisted of healthcare providers working at the sampled facilities, who were on duty during the data-collection period.

**TABLE 2:** Participants: Healthcare workers.

Code	Category	Age	Facility	Years of experience
HCP1	Community healthcare worker	29	Facility B	3
HCP2	Peer educator	23	Facility A	10
HCP3	Lay counsellor	31	Facility A	4
HCP4	Community healthcare worker	40	Facility C	7
HCP5	Lay counsellor	43	Facility B	4
HCP6	Peer educator	38	Facility C	4
HCP7	Professional nurse	42	Facility C	6
HCP8	Professional nurse	48	Facility C	6
HCP9	Professional nurse	28	Facility B	3

Note: All participant were female.

### Approaches to promote condom use

The study participants indicated that the approaches aimed at improving condom use were linked to information sharing, with the subthemes Education, Educational materials on condom use, and a Multi stakeholder approach to promoting condom use. The identified subthemes are discussed in the following subsection.

#### Education

The healthcare providers who participated in the study indicated that they offer health education when visiting communities, to explain the risks associated with non-condom use. One participant commented as follows:

‘We also talk about teenage pregnancy, and [*the*] disadvantages of engaging in unprotected sex, and what might happen. If they engage in sexual activities, we explain that they can contract diseases such as STIs [*sexually transmitted infections*], falling pregnant before time, while still very young, and teenage pregnancy.’ (HCP1, Facility B, 29 year old)

‘We do door to door, entering all houses and inviting people to attend, [*we*] create space to explain the value of using condoms.’ (HCP2, Facility A, 23 years old)

‘We show them different condoms and also how they are used, because if they fail to use […] condoms, there are negative consequences.’ (HCP4, Facility C, 40 years old)

This view was, however, not supported by one of the young adult participants, who was on ARTs and came to collect medication from one of the facilities every month. That participant indicated that no health education on condom use was offered. She admitted that she did not know how to use a female condom, because she had never been given information or taught how to use it. She said the following:

‘I come here every month, but I have never been given health education about condom use or safe sex. They say nothing about condom use. I used to hear nurses teaching us when I was still taking my treatment at the hospital, not here.’ (P7, Facility B, 24 years old)

In supporting the claim by the young adult, one HCP who was a lay counsellor indicated their lack of adequate information to share with the communities when going on community outreach. She verbalised:

‘We also want to attend workshops because in workshops, we are taught about things that we can share when we go out in communities, sometimes we are asked questions by patients. Patients can ask you questions that will frustrate you, and you find yourself not knowing how to respond, but if we are taught these things then one can provide a response that makes sense.’ (HCP 3, Facility A, 31 years old)

#### Availability of educational materials on condom use

One of the interview guide questions for healthcare providers sought to determine the availability of educational materials or pamphlets relating to HIV prevention, including guidelines on condom use. All the participants indicated the non-availability of such materials across all sampled facilities. As one participant explained:

‘Unfortunately, educational materials [such] as pamphlets are not available. It has been two years now, without them. Previously we used to have all the types of pamphlets, for example HIV [*human immunodeficiency virus*]-related, and […] ones for TB [*tuberculosis*], STIs [*sexually transmitted infections*], but now there is a serious shortage. They used to bring pamphlets for everything, and the consultation table and the drawers would be full, and one would decide to issue to patients depending on the need, but now it is very dry. I was discussing this matter with the operational manager last week, because we will [*be*] having an HIV awareness campaign, but we don’t have even one pamphlet. The operational manager said she went to search for pamphlets at the district, but came back with nothing. The one that we commonly have is for TB – the pamphlets you are talking about, we do not have it.’ (HCP 7, Facility C, 42 years old)

#### Multi sectoral team approach

During data collection at the sampled facilities, the researcher found that a multidisciplinary team approach was already being implemented in respect of HIV and AIDS prevention and health promotion, management, treatment and care, although a further strengthening and integration of activities was required. The quotes reflected here suggest that healthcare providers (nurse practitioners and lay counsellors) are involved in one way or another in promoting condom use.

The participating nurses indicated that they educated clients on the importance of condom use and demonstrated how a condom (especially the male condom) is used. Lay counsellors professed to be providing pre- and post-counselling when doing HIV testing, whether it was provider- or client-initiated, and indicated that they always emphasised the importance of condom use. As one healthcare participant stated:

‘Yes, they are taught by lay counsellors. They teach them and also demonstrate how condoms are utilised. They are educated by nurses, social workers. Other stakeholders also participate.’ (HCP8, Facility C, 48 years old)

Peer educators are responsible for distributing condoms to hotspots such as taverns or shebeens, and also to sex workers in the community. As one participant noted:

‘… [*I*]t is […] amazing [*to*] me. I cannot know why, when condoms are distributed all over and […] given to people, and [*despite*] having peer educators of sex workers who also distribute the condoms, they are still not used.’ (HCP2, Facility A, 23 years old)

### Reasons for condom use

The second superordinate theme covered the reasons for condom use. The theme in this section was self-protection and fear of consequences of no condom use during sexual contact, with the following subthemes: Prevention of diseases, Prevention of pregnancy, and Not trusting a sexual partner. Each subtheme is discussed in greater detail in the following subsection.

#### Prevention of diseases

Condom use is seen as a critical component of any comprehensive and sustainable approach to prevention of STIs.^16^ The main reason for condom use amongst HIV-positive clients on ART therapy is to prevent transmission of new viral strains and other STIs. A strong focus of the revised National Strategic Plan on HIV and AIDS and TB, 2017–2022 is to work towards the prevention of HIV infection amongst adolescent girls and young women, because of the higher rates of infection in these population groups.^17^ Early infection with HIV amongst teenage girls and many young women negatively and irreversibly affect their livelihoods from their teens onwards.^17^ In this study, almost all participants understood the rationale for using condoms, be it to prevent HIV or AIDS, teenage pregnancy or STIs. As two young participants observed:

‘… [*P*]revention of diseases such as HIV [*human immunodeficiency virus*] […], “u wela” [*a sexually transmitted infection*] and STIs [*sexually transmitted infections*]. It is when [*a*] female have backstreet abortion abort[*s*] and ha[*s*] sex with me while still [*being*] dirty [*unclean*]. In reality, “u wela” happens when a female has [*had*] an abortion, and this happens mostly if it is [*a*] backstreet abortion.’ (P3, Facility A, 24 years old)

‘It’s good advice, because if young adults don’t use condoms, they will get HIV or [*an*] STD [*sexually transmitted disease*]. We are using condoms to protect our CD4 [*cluster of differentiation 4*] count.’ (P7, Facility A, 24 years old)

#### Prevention of pregnancy

According to the participants, condoms offer an effective barrier against both teenage and unplanned pregnancies. As two of the participants indicated:

‘When I hear people talking, they say in that place, people undress while dancing. A lot of youth are found there. People go there with alcohol and drugs. It means that they are engaging in sexual activities without protection and end up with teenage pregnanc[*ies*]. So, if they use condoms, they will not fall pregnant.’ (HCP 1, Facility B, 29 years old)

‘If they engage in sexual activities, we explain that they can fall pregnant before time, while still very young. Condoms protect […] children from falling pregnant while they are still young and at school.’ (HCP3, Facility A, 31 years old)

One of the male participants, a university student, indicated that he does not want to make a girl pregnant when he is still studying, as he is not yet ready to assume that kind of responsibility. He commented:

‘The other reason is to avoid things which one is not prepared for, such as having impregnated a girl. I am preventing such things.’ (P2, Facility A, 23 years old)

#### Prevention of ‘u wela’

‘U wela’ is a belief, predominantly in traditional Venda cultural settings, that if a man has sex with a girl who was pregnant and had a backstreet abortion, he will be infected, become very sick and possibly even die. The reasoning is that the woman at that stage is very contagious, because she is not ‘clean’, as would be the case where a termination of pregnancy is conducted in a hospital setting. The reasons for condom use, as indicated by two male participants, is to prevent ‘u wela’, as this comment indicates:

‘… [*I*]t is the prevention of disease[*s*] like “u wela” and STDs [*sexually transmitted diseases*]. “U wela” frequently happens if a backstreet abortion is done. If a person has aborted in the western way, they are usually cleaned. It is when the female abort[*s*] and ha[*s*] sex with me while still “dirty”, because of leftovers from [*the*] aborted baby in the womb.’ (P3, Facility A, 24 years old)

#### Not trusting a sexual partner

The issue of trust was raised during data collection as a predictor of condom use, especially by male participants. This is consistent with other research findings, for instance those reported by Gutierrez, Pinto, Basso, Spiassi, Lopes and Barros,^18^ which revealed that trust in a partner is a serious challenge. Here are the views of several study participants in this regard:

‘We were not trusting each other, thinking that maybe one might have […] **AIDS** and [*an*] STI [*sexually transmitted infection*].’ (P5, Facility B, 19 years old)

‘One can see that if you have sex with this one without a condom, you will be killing yourself. Those whom I do not doubt, I do not use condoms.’ (P8, Facility B, 20 years old)

‘Maybe it’s their boyfriend who advise[s] them not to use condoms. Maybe this boy is [*too*] controlling, they tell them that it means “you don’t love me”, “you don’t trust me” if you want us to use a condom.’ (P8, Facility B, 20 years old)

## Discussion

The participants in this study were nine young adults aged 18–24 years, who had visited the primary healthcare facilities in question for services during the data-collection period, and nine healthcare workers who were available at the same time, in the sampled primary healthcare facilities. The participants shared approaches that could be used to promote condom use amongst young adults, who are regarded as a vulnerable group for HIV or AIDS infection.^18,19,20^ The participating healthcare workers had more than three years’ experience working with young adults in primary healthcare and community settings. Kanda and Mash^21^ reveal that a lack of information about condoms and condom use is one reason for the failure to use condoms. One of the five pillars in ensuring coverage of the 90-90-90 strategy (90% of population HIV tested, 90% of HIV-positive are sustained on antiretroviral (ARV) treatment and 90% of HIV patients on ARV have viral suppression) , as contained in the 2016 Gap Report,^22^ includes a combination of prevention strategies, such as comprehensive sexuality education, economic empowerment, and access to sexual and reproductive health services for young women and adolescent girls and their male partners in high-prevalence locations. In support of this view, a lack of health education was a general concern at all the sampled facilities. The researcher found that lay healthcare providers had access to limited information, and were therefore not able to provide clients with details they themselves did not know.

The participants reported that information sharing was key to promoting condom use amongst the youth. The young clients who were collecting medication from the primary healthcare facilities in question denied that healthcare workers had offered them advice on condom use. They mentioned that, despite repeated visits to the healthcare facilities, they were never taught about the use of, or informed about, condoms. Lay counsellors’ limited information reported in this study, could have been the obstacle in promoting condom use to some young adults who were serviced and not advised on condom use. The reflection above is corroborated by Lelaka,^23^ which brought to light the challenges faced by lay counsellors. The study revealed that lay counsellors reported limited information, and when patients come and ask questions, they cannot answer because they also do not understand the question themselves. This is further supported by Cumber and Tsonga-Gwegweni,^24^ who found that street children in Cameroon knew about condoms but they did not have sufficient information regarding the importance of regular use of condoms.

Contradicting the young adults’ experiences of lacking education on condom use, the healthcare providers reported sharing information on the disadvantages of having unprotected sex, preventing teenage pregnancies, the value of condom use and how to use different condoms. This blaming game could be avoided if educational materials were available and healthcare providers were attending regular workshops on health promotion to improve their skills in health education. This finding is supported by that of researchers such as Arian, Kamali, Tabatabaeichehr and Arashnia,^25^ who posit that educational materials are of utmost importance in health promotion systems, as they direct patients’ learning processes and offer additional information on individual conditions. In this regard, all clinics should have printed materials that promote condom use as an essential component of any HIV or AIDS, STI and teenage pregnancy prevention strategy. Regular in-service training or workshop should be conducted with healthcare providers to keep them up to date with health promotion issues.

The lack of information amongst young adults in this study was compounded by the absence of educational materials such as pamphlets relating to HIV prevention or condom use. These are important instructional tools that provide much-needed information to young adults and can improve their health outcomes if they are already living with HIV or AIDS. At the three sampled facilities there were no pamphlets or educational materials pertaining to HIV or AIDS, or condom use for health promotion or prevention. To provide quality information related to condoms, there must be adequate resources, including, amongst others, educational materials or leaflets that can extend or enhance the health education provided face to face. Both groups of participants confirmed the scarcity of brochures on condom use.

A multisectoral team approach was suggested as being the best way of promoting information on condom use, because different team members could share the burden of patient care. Coordinating a multisectoral team approach could be helpful in promoting condom use, which is a notion supported by Ojikutu, Holman, Kunches, Landers, Permutter, Ward, Fant and Hirschhorn,^26^ who insist that HIV remains a challenge that requires a multidisciplinary, collaborative and comprehensive approach, to ensure optimal outcomes by both clinical and nonclinical providers.

The study participants were very much aware of the need to prevent the spread of disease and unplanned pregnancies through condom use. Most participants verbalised that they support the use of condoms fearing the consequences of non-use of condoms. When asked why they advocate using condoms, all participants confirmed that it prevented pregnancies and the spread of STIs. This finding concurs with that of Muswede and Mavhandu,^27^ Ajayi et al.,^20^ and Madiba and Mokgatle,^19^ who found that condoms are considered useful in preventing unplanned pregnancies and STIs, including HIV or AIDS. According to the Health Belief Service model theory framework,^28^ young female adults who perceive themselves as being at high risk of engaging in unprotected sex, allow certain cues to guide their actions and require less persuasion to use condoms to protect themselves. By contrast, individuals who believe they are at low risk of exposure require more intense external cues before seeking advice from a health service provider.

The participants revealed that preventing ‘u wela’ could be achieved through condom use. These views are consistent with those reported in a study by Bourbonnais^29^ in Kenya, which revealed that women continue to turn to unsafe procedures for terminating pregnancies. This was attributed to a lack of awareness of their rights, stigmas about abortion, and healthcare workers’ resistance. The study further revealed that complications from unsafe abortions are estimated to account for one-third of maternal deaths in Kenya.

Lack of trust in sexual partners reportedly prompted condom use, particularly amongst males. This is consistent with the findings of Fehr, Vidourek and King,^30^ as well as Wet-Billings and Billings,^31^ who found that men are more likely to use condoms when they do not trust a partner to be faithful. Distrust in relationships and gender inequality are cited in the literature^21,29^ as predictors of condom use. One of the male participants in this study indicated that his decision whether or not to use condoms depended on the type of girl he met. Only if he had doubts about her (fidelity), did he use condoms. The same sentiments were common amongst participants in stable, monogamous relationships.

## Limitations of the study

The study was limited to three primary healthcare facilities in Vhembe district in Limpopo province. Therefore, the findings reported on here are applicable only to healthcare providers and young adults from the district under study. The motives for condom use were similar to those established in other regions and countries as reported in the literature. As the study was qualitative in nature, and employed an exploratory qualitative design, data collection was carried out over a relatively short period. Adopting a longitudinal approach would expand the scope of the study.

## Implications of the study

The researchers posit that this is a very important study, especially in the South African context, because anecdotal evidence from related studies, including by Muswede and Mavhandu-Mudzusi^27^ suggest improving the attitudes of healthcare providers and promoting the correct and consistent use of condoms as strategies for addressing the failure to use condoms amongst young adults in the Vhembe district. These findings will guide healthcare workers to bring about transformation within that specific context.^32^ Knowledge of the reasons for condom use/non-use might improve uptake as a whole amongst young adults.

## Recommendations

Awareness campaigns amongst young adults should focus on promoting condom use, including a demonstration of how to use a condom (for males or females).

*Imbizo*s should be conducted, in collaboration with traditional leaders in the community, on all matters related to health promotion and disease prevention, including the importance of condom use. These initiatives should include clinic committee members helping to mobilise and disseminate information in the community to achieve a heightened impact.

The healthcare team should target hotspots such as taverns and shebeens to educate young adults on the importance of condom use.

Registers should be kept, detailing all ward-based activities, health promotion and disease prevention initiatives, the topics covered and the participants who attended.

A record of learners’ attendance of health education sessions should be available in the PHC facilities and be signed by the staff member who provided the training.

Comprehensive educational materials or pamphlets focusing on condom use as part of HIV or AIDS prevention initiatives must be made available to the public to provide additional information to young adults and their sexual partners.

All primary healthcare clinics must implement *vhaswaphanda* (youth first) principles and adopt a youth-friendly service approach to increase the uptake and use of condoms amongst young adults.
